# Complete Genome Sequence of a Parabacteroides distasonis Strain (CavFT hAR46) Isolated from a Gut Wall-Cavitating Microlesion in a Patient with Severe Crohn’s Disease

**DOI:** 10.1128/MRA.00585-19

**Published:** 2019-09-05

**Authors:** Falong Yang, Anand Kumar, Karen Walston Davenport, Julia Mae Kelliher, Jessica C. Ezeji, Caryn E. Good, Michael R. Jacobs, Mathew Conger, Gail West, Claudio Fiocchi, Fabio Cominelli, Armand Earl Ko Dichosa, Alexander Rodriguez-Palacios

**Affiliations:** a Division of Gastroenterology & Liver Disease, Case Western Reserve University School of Medicine, Cleveland, Ohio, USA; b Digestive Research Health Institute, Case Western Reserve University School of Medicine, Cleveland, Ohio, USA; c B-10, Biosecurity and Public Health, Los Alamos National Laboratory, Los Alamos, New Mexico, USA; d Department of Pathology, University Hospitals Cleveland Medical Center, Cleveland, Ohio, USA; e Department of Pathology, Case Western Reserve University, Cleveland, Ohio, USA; f Department of Inflammation and Immunity, Lerner Research Institute, The Cleveland Clinic Foundation, Cleveland, Ohio, USA; g Digestive Health Institute, University Hospitals Cleveland Medical Center, Cleveland, Ohio, USA; University of Maryland School of Medicine

## Abstract

Crohn’s disease (CD) is a chronic inflammatory bowel disease (IBD) of the digestive tract in humans. There is evidence that Parabacteroides distasonis could contribute to IBD. Here, we present the complete genome sequence of a strain designated CavFT-hAR46, which was isolated from a gut intramural cavernous fistulous tract (CavFT) microlesion in a CD patient.

## ANNOUNCEMENT

Crohn’s disease (CD) is a chronic inflammatory bowel disease (IBD) of the digestive tract in humans that affects all layers of the gut wall. Although CD is not primarily caused by bacteria, there is evidence that gut microbiome alterations are associated with CD (1, 2). However, it remains unclear which, and how, microbes worsen CD. We recently isolated Parabacteroides distasonis (NCBI taxid 823, confirmed by 16S rRNA gene Sanger sequencing and matrix-assisted laser desorption ionization–time of flight [MALDI-TOF] mass spectrometry) from an intramural cavernous fistulous tract (IM-CavFT) (3) microlesion from the gut wall of a patient who underwent surgical removal of the ileum as treatment for CD. The surgical specimen was obtained as a deidentified sample via the Biorepository Core of the NIH Silvio O. Conte Cleveland Digestive Disease Research Core Center (Cleveland, OH, USA), which obtained the consent and approval from surgical patients following protocols approved by the Case Western Reserve University and University Hospitals Cleveland Medical Center institutional review boards.

Parabacteroides distasonis (NCBI lineage: bacteria; FCB group; *Bacteroidetes*/Chlorobi group; *Bacteroidetes*; *Bacteroidia*; *Bacteroidales*; *Tannerellaceae*; *Parabacteroides*) has been reported to be a beneficial commensal gut microorganism (4–6); however, evidence indicates that P. distasonis could contribute to IBD (7, 8). Herein, we announce the complete genome sequence of a strain we designated CavFT-hAR46 (pronounced “cavftar46”) since its human origin in a gut wall lesion could help identify potential pathogenic mechanisms of bacterial invasion, intramural lesions, and complications in CD.

This strain was isolated anaerobically from IM-CavFT debris which was obtained by microdissection of a fresh specimen, as we previously reported (3). The microscopically dissected debris was then plated onto and purified using prereduced tryptic soy 5% defibrinated sheep blood agar (80% N–10% H–10% CO_2_, at 37°C; Thermo Fisher Scientific) using a variable-atmosphere anaerobic Whitley workstation A85 (540 plate capacity; Microbiology International, Inc.). Individual colonies were subcultured and purified in the same agar and then banked in prereduced brain heart infusion (BHI) broth with 7% dimethyl sulfoxide (DMSO). DNA was extracted from the original isolate using the MagAttract high-molecular-weight (HMW) DNA kit (Qiagen).

Genome sequencing was conducted using Pacific Biosciences (PacBio) reagents (DNA Link, Inc., South Korea). The SMRTbell library was constructed with the SMRTbell template prep kit 1.0 (product number 100-259-100; PacBio), with removal of small fragments (<20 kb) using the BluePippin size selection system. The constructed library was validated using an Agilent 2100 bioanalyzer and sequenced using one single-molecule real-time (SMRT) cell, P6-C4 chemistry (DNA sequencing reagent 4.0), and PacBio RS II 240-minute movies (9). Default parameters were used for all software, unless otherwise specified. We produced 128,581 long reads and 1,132,591,489 bp after subread filtering. *De novo* assembly was conducted using the Hierarchical Genome Assembly Process (HGAP, version 2.3) (10), including consensus polishing with Quiver. Since the average sequencing coverage was 168× for a total of 5,079,886 bp distributed across 5 contigs, we performed error correction based on the longest (150,079,410 bp) seed bases (~30× coverage) and then assembled them with error-corrected reads (contig *N*_50_, 4,988,677 bp for a 5 total contigs with a length of 5,079,886 bp). To examine the circularity form of the contigs, we used MUMmer 3.5 and trimmed one of the self-similar ends for manual genome closure using previously described protocols (10, 11). Combined sequencing produced one polished single circular contig with no gaps or plasmids.

For contextualization, we used the Pathosystems Resource Integration Center (PATRIC v.3.5.38) genome database for automated annotation and overall comparison to 49 other P. distasonis sequenced strains (1 May 2019; https://patricbrc.org/; 99% DNA purity, free of other bacterial contaminants). Because most strains (*n* = 47) were whole-genome shotgun (WGS) sequenced and/or had multiple contigs (range, 3 to 1,920), we used the 1-contig P. distasonis reference genome/strain (ATCC 8503) and three other strains to conduct comparative single-nucleotide polymorphism (SNP) analysis of our contig using EDGE Bioinformatics (12, 13). Collectively, our strain matched the ATCC 8503 reference genome at ∼85% identity. The other strains matched at 80% (Parabacteroides sp. strain CT06) or <10% (Table 1).

**TABLE 1:** Genomic features of Parabacteroides distasonis strain CavFT-hAR046 and single nucleotide polymorphism differences compared to other complete *Parabacteroides* genomes

Feature^*a*^	Data for strain:			
	CavFT-hAR46	ATCC 8503	CT06	82G9
Location of origin (yr)	Ohio, USA (2017)	USA	South Korea	Japan
Accession no.	CP040468	NC_009615	NZ_CP022754	NZ_LR215978
Genome size (bp)	4,952,323	4,811,379	5,372,666	5,212,259
No. of CDS^*b*^	4,263	4,216	4,686	4,365
G+C content (%)	45.2	45.1	45.2	45.2
No. of rRNAs	21	21	21	21
No. of proteins with functional assignments	2,694	2,639	2,875	ND^*c*^
No. of hypothetical proteins	1,569	1,487	1,811	ND
No. of antibiotic resistance genes	24	22	28	ND
No. of genomic polymorphisms of CavFT-hAR046 strain compared to ATCC 8503, CT06, 82G9 strains				
Synonymous SNPs	NA^*d*^	10,600	14,748	ND
Nonsynonymous SNPs	NA	3,443	4,986	ND
Indels	NA	478	689	ND

a Basic genome features in this table were derived from pairwise analysis using PATRIC annotation definitions.

b CDS, coding sequences.

c ND, not determined.

d NA, not applicable.

In contrast to two previously available complete genomes of P. distasonis (ATCC 8503 and 82G9 in Table 1) and strain NBRC 113806, which seemed to have been isolated from feces, herein, we report the complete genome sequence of a potential pathogenic strain with relevance to chronic forms of intestinal inflammation, especially to severe and incurable surgical forms of CD. Since this strain was isolated from chronic inflammatory lesions present in the gut wall of CD patients, we expect that future functional microbiological and genetic analyses with this strain could help determine the role that intestinal and invasive bacteria play in IBD. This is the lead genome of a series of potential pathogenic bacteria we have identified in Crohn’s disease.

### Data availability.

This genome sequence has been deposited in GenBank under the accession number CP040468. Its associated BioProject, BioSample, and Sequence Read Archive (SRA) numbers are PRJNA542869, SAMN11642307, and SRR9221602, respectively. For public availability as gold standard, this genome has been annotated by the National Center of Biotechnology Information using the latest Prokaryotic Genome Annotation Pipeline which uses new hidden Markov models for antimicrobial resistance proteins, and curated complex domain architectures for functional annotation of proteins (assembly name ASM614918v1, RefSeq sequence NZ_CP040468.1; www.ncbi.nlm.nih.gov/genome/annotation_prok/).
